# Supplementary material for: Different trajectories of adolescent mental health problems before and over the course of COVID-19: Evidence of increase, decrease, and stability
Source: PLOS Ment Health. 2025 May 6;2(5):e0000292. doi: 10.1371/journal.pmen.0000292 (PMC12798380; doi:10.1371/journal.pmen.0000292)
Supplement: S1 Checklist — (DOCX) [file pmen.0000292.s001.docx]

**S1 Checklist: GRoLTS Checklist - Guidelines for reporting on latent trajectory studies**

**1. Is the metric of time used in the statistical model reported?**

“The intervals between time points were adjusted to correspond with the time period between the measurements (i.e., the time points for the linear slope factor were fixed at 0, 0.5, 1, and 2).” (p. 10)

**2. Is information presented about the mean and variance of time within a wave?**

“The mean time interval between T1 and T2 was 6.56 months (*SD* = 1.15), between T2 and T3 was 5.10 months (*SD* = 0.77), and between T3 and T4 was 12.64 months (*SD* = 1.42).” (p. 7)

However, within-wave variability was not accounted for in the models due to estimation problems that arose when incorporating random factor loadings with individually varying times intervals.

**3a. Is the missing data mechanism reported?**

“Little’s chi-square test for missing data indicated that the missing data on the mental health problems subscales were not completely missing at random, χ^2^ (144) = 191.52, *p* = .005.” (p. 11)

**3b. Is a description provided of what variables are related to attrition/missing data?**

“Adolescents who participated in all waves reported more emotional symptoms at T1 than adolescents who did not participate in all waves (*M* = 3.43 vs. *M* = 3.00), and less often had a migration background (15% vs. 27%). We did not find differences in conduct problems, hyperactivity‐inattention problems or peer relationship problems at T1, gender and family SES between those adolescents who participated in all waves and those who did not.” (p. 11-12)

**3c. Is a description provided of how missing data in the analyses were dealt with?**

“To handle missing data without deleting cases and to limit the bias associated with missing data as well as to account for the non-normality of the outcome variables, we used maximum likelihood with robust standard errors (MLR).” (p. 12)

**4. Is information about the distribution of the observed variables included?**

See the answer to the previous question. The answer is the same as for item 3c, as MRL also addresses non-normality of the outcome variables.

**5. Is the software mentioned?**

“To investigate adolescent mental health trajectories before and during the pandemic, we conducted latent class growth analysis (LCGA) using Mplus version 8.8.” (p. 10)

**6a. Are alternative specifications of within-class heterogeneity considered (e.g., LGCA vs. LGMM) and clearly documented? If not, was sufficient justification provided as to eliminate certain specifications from consideration?**

“We selected LCGA over latent growth mixture modeling because our primary interest was to identify subgroups with similar patterns, rather than to model individual differences within those subgroups.” (p. 10)

**6b. Are alternative specifications of the between-class differences in variance–covariance matrix structure considered and clearly documented? If not, was sufficient justification provided as to eliminate certain specifications from consideration?**

“To avoid convergence issues, the residual variances and the variance-covariance matrix were fixed across latent classes.” (p. 11)

**7. Are alternative shape/functional forms of the trajectories described?**

“The Satorra-Bentler scaled chi-square difference test was executed to test whether a linear or a quadratic growth model better fitted the data.” (p. 10-11)

In the Results section, we reported the quadratic growth model in comparison to the linear growth model for each mental health indicator.

**8. If covariates have been used, can analyses still be replicated?**

“After estimating the best fitting model and establishing the number of classes, we used a three-step approach to examine the extent to which the identified classes differed from each other on the demographic and social support variables. First, the latent class model was estimated. Second, the most likely class variable was created using the latent class posterior distribution. Third, we added the demographic and social support variables one by one and tested the equality of means or percentages across classes using chi-square tests. In this third step, misclassification from the second step was taken into account.” (p. 11)

**9. Is information reported about the number of random start values and final iterations included?**

For the single-class growth models we reported: “We used starting values 100 for the single-class growth models.” (p. 10)

For the LCGA models we reported: “We used starting values 100 10 for these models. If we encountered computation problems, we increased the LRTSTARTS to 500 100 250 50.” (p. 11)

**10. Are the model comparison (and selection) tools described from a statistical perspective?**

“The number of classes was established based on a trade-off between fit indices, parsimony, and interpretability. The best fitting model had the lowest Bayesian Information Criteria (BIC) and a significant improvement in model fit compared to a model with one class less based on the Lo-Mendell-Rubin adjusted Likelihood Ratio Test (LMR-LRT) and Bootstrap Likelihood Ratio Test (BLRT). Additionally, the classes needed to have a sufficiently large size (n > 50) and be substantively interpretable as distinguishable classes.” (p. 11)

**11. Are the total number of fitted models reported, including a one-class solution?**

Table 2 provides an overview of the model fit indices for a one-class solution to a four-class solution for peer relationship problems, a five-class solution for conduct problems, and a six-class solution for hyperactivity-inattention problems.

**12. Are the number of cases per class reported for each model (absolute sample size, or proportion)?**

Table 3 provides information on the number of individuals assigned to each latent class in the final model. Additionally, Table 2 presents the minimum class size for each LCGA model.

**13. If classification of cases in a trajectory is the goal, is entropy reported?**

Table 2 provides the entropies for the final class solutions.

**14a. Is a plot included with the estimated mean trajectories of the final solution?**

Figure 1 includes plots of the final class solutions for the different mental health indicators.

**14b. Are plots included with the estimated mean trajectories for each model?**

Plots with the estimated mean trajectories for each model are available on OSF (<https://osf.io/j2t6w/>).

**14c. Is a plot included of the combination of estimated means of the final model and the observed individual trajectories split out for each latent class?**

Plots with the estimated means of the final model and the observed individual trajectories for each latent class are available on OSF (<https://osf.io/j2t6w/>).

**15. Are characteristics of the final class solution numerically described (i.e., means, SD/SE, n, CI, etc.)?**

Table 3 presents the results for the final class solutions, including means/proportions, standard deviations, and sample sizes. Asterisks indicate whether mean/proportion is significant at an alfa level of .05, .01 or .001.

**16. Are the syntax files available (either in the appendix, supplementary materials, or from the authors)?**

The dataset and syntax are available on OSF (<https://osf.io/j2t6w/>).
