# Supplementary material for: Different trajectories of adolescent mental health problems before and over the course of COVID-19: Evidence of increase, decrease, and stability
Source: PLOS Ment Health. 2025 May 6;2(5):e0000292. doi: 10.1371/journal.pmen.0000292 (PMC12798380; doi:10.1371/journal.pmen.0000292)
Supplement: Attachment — Submitted filename: Response to Reviewers.docx [file pmen.0000292.s004.docx]

**Editor**

Thank you for submitting your manuscript "Trajectories of adolescent mental health problems before and during the COVID-19 pandemic" to PLOS Mental Health. We have now received comments from two expert reviewers, and I am pleased to say that both find your study valuable and methodologically sound. They appreciate the large sample size, longitudinal design, and consideration of various demographic factors. However, they have identified several areas where revisions could enhance the manuscript's quality and impact.

Based on the reviewers' comments, I would like to request the following revisions:

Methodology and Reporting:

Please follow the GRoLTS-checklist guidelines for reporting latent trajectory studies, as suggested by Reviewer 1. This will ensure comprehensive reporting of your analytical choices.
Provide clearer justification for your analytical decisions, particularly regarding the latent class growth analysis.
Consider using a three-step approach for analyzing the impact of auxiliary predictors on class membership, as detailed by Reviewer 1. This method would account for classification uncertainty and potentially yield more robust results.

Results Interpretation:

Contextualize the observed changes in mental health scores with established severity thresholds for the SDQ. This will help readers understand the clinical significance of the identified trajectories.
Explore possible explanations for why some adolescents showed improved mental health during the pandemic, as suggested by Reviewer 2.

Discussion Enhancement:

Deepen the theoretical exploration of your findings, particularly regarding gender differences and the lack of consistent associations with demographic characteristics.
If applicable, consider discussing the functional impact supplement of the SDQ.

Minor Clarifications:

Provide more precise definitions for certain terms (e.g., "migration background") and age ranges.
Strengthen the introduction by emphasizing the importance of long-term trajectories and distinguishing between peer socialization and formal mental health services.
Both reviewers believe your manuscript has the potential for publication in PLOS Mental Health after addressing these concerns. Please carefully consider all suggestions, particularly those related to methodology and result interpretation, to strengthen your paper's contribution to the field.

Dear Dr. Gareth,

Thank you very much for inviting us to revise and resubmit our manuscript, entitled “Different trajectories of adolescent mental health problems before and over the course of COVID-19: Evidence of increase, decrease, and stability” (PMEN-D-24-00443). My co-authors and I greatly appreciate your and the reviewers’ thoughtful and constructive comments, which were valuable in improving our manuscript. We revised the manuscript, among other issues, we 1) incorporated the items from the GRoLTS-checklist, 2) added information about the clinical significance of the increasing trajectories in particular, and 3) elaborated on our findings in the discussion section using relevant theoretical frameworks. Below, we describe in detail how we addressed each of the comments, and how we incorporated the comments into the revised version of the manuscript. We are confident that the overall quality of the manuscript has improved as a result of these revisions.

We hope that you will find the modifications satisfactory, and we are looking forward to hearing from you.

In advance, thank you very much for your attention to our work.

Your sincerely, on behalf of all authors,

Coriena de Heer

**Journal requirements**

1. Please amend your detailed Financial Disclosure statement. This is published with the article. It must therefore be completed in full sentences and contain the exact wording you wish to be published.

Response: Our Financial Disclosure statement is as follows: This work was supported by the IMPROVA Project, which received funding from the European Union's Horizon Europe Research and Innovation Programme under Grant Agreement No. 101080934. The funder had no role in the study design, data collection and analysis, decision to publish, or preparation of the manuscript.

2. Thank you for uploading your study's underlying data set. Unfortunately, the repository you have noted in your Data Availability statement does not qualify as an acceptable data repository according to PLOS's standards.

Response: We made the dataset and syntaxes available through the Open Science Framework (OSF) repository (<https://osf.io/j2t6w/>). In the PLOS list of recommended repositories, OSF is included as a cross-disciplinary repository. In accordance with PLOS standards for data deposition, we applied the CC BY license and made the OSF folder publicly accessible as of November 8, 2024.

**Reviewer 1**

Overall, the manuscript presents a methodologically robust study with sound findings. The conclusions are well-framed and supported by the data, with appropriate consideration of the study's limitations and ethical context. Further theoretical exploration could enhance the interpretation, but the research offers valuable insights into the complex mental health trajectories of adolescents during the COVID-19 pandemic. The statistical analysis is rigorous, effectively capturing changes in adolescent mental health over time. The large sample size and consideration of demographic factors provide a strong foundation for the conclusions, despite some limitations in generalisability.

The authors have made the dataset available as stated: "The dataset and syntaxes are available in the OSF database (https://osf.io/j2t6w/). We will make the OSF folder public after the manuscript is accepted."

Response: We would like to thank Reviewer 1 for the kind words about our manuscript, and suggestions to improve the manuscript. Below we will address each of the comments and suggestions.

Minor Revisions:

1. Introduction: When discussing the importance of peer and formal support, it would be beneficial to differentiate between peer socialisation and formal mental health services. This distinction could enrich the argument that both were disrupted during the pandemic, e.g., "Both peer socialisation and formal mental health services, vital for adolescent well-being, were significantly disrupted due to school closures and social distancing measures."

Response: We revised the text to more clearly differentiate between peer socialization (peer interactions) and formal mental health services. The text now reads:

“Both interactions with peers and access to formal mental health services within school, which both are important for adolescent mental health [1,2], were significantly disrupted due to lockdowns, school closings, and social distancing measures.” (p. 3)

2. Introduction: While the section on mental health trajectories is thorough, emphasising the importance of examining long-term trajectories would strengthen the argument. A brief note on how understanding these trajectories can inform interventions more effectively than short-term studies could be added.

Response: Following the suggestion of Reviewer 1, we now elaborated on the importance of examining long-term trajectories.

“Addressing the limitations of previous research, this study assessed mental health problems in a large sample of adolescents before the pandemic and three times during the first eighteen months of the pandemic. Examining trajectories over a longer time period provides a deeper understanding of how adolescent mental health has developed during this critical period and therefore offers more valuable insights for interventions targeting mental health problems than short-term studies. This deeper understanding is necessary to determine the necessity of interventions to maximize their impact and be tailored to adolescents’ needs, circumstances, and readiness to engage in these interventions. For example, adolescents go through significant changes, and some issues may be developmentally normal or typical within the context of a national pandemic (e.g., anxiety). Longitudinal data allow us to examine whether behaviors and feelings persist or worsen over time, which can help improve the effectiveness of interventions.” (p. 5)

3. Methods: The age range at T1 is presented as an average with a standard deviation, but specifying the actual age range (e.g., 15-21) would clarify the group being studied.

Response: We added the age range to the Method section:

“The mean age of the participants at T1 was 17.91 years (*SD* = 2.05, range: 15.12-31.04)” (p. 8)

4. Methods: A clearer definition of "migration background" would enhance clarity, such as: "Participants were considered to have a migration background if at least one parent was born outside the Netherlands."

Response: Although this information was included in the coding of migration background, we agree that providing a more explicit definition of migration background is informative for the reader, and we have followed the reviewer’s suggestion.

“The mean age of the participants at T1 was 17.91 years (*SD* = 2.05, range: 15.12-31.04), 41.0% was boy, and 25.3% had a migration background. Participants were considered to have a migration background if at least one parent was born outside the Netherlands.” (p. 8)

5. Methods: Providing a brief rationale for selecting latent class growth analysis (LCGA) could add depth.

Response: We have added the following rationale in the Method section:

“We selected LCGA over latent growth mixture modeling because our primary interest was to identify subgroups with similar patterns, rather than to model individual differences within those subgroups.” (p. 10)

6. Discussion: While the discussion acknowledges that some adolescents exhibited resilience while others struggled, exploring why some experienced decreases in conduct problems or hyperactivity-inattention during the pandemic could strengthen the conceptual implications. Linking this to developmental or psychological theories (e.g., the stress-buffering model of social support) would deepen the analysis.

We agree with the reviewer that it is valuable to underpin the findings with a theoretical framework. However, given our findings, we are uncertain whether the stress-buffering model of social support is the most appropriate theory. Specifically, we found that family and friend support were associated with a decrease in hyperactivity-inattention problems, but not with conduct problems. Another theory that could help explain why some adolescents show a decrease in conduct problems or hyperactivity-inattention is the Self-Determination Theory, which we now integrated into the Discussion:

“The decreases in conduct problems and hyperactivity-inattention problems might be attributed to the increased time adolescents spent at home during lockdowns. From the perspective of Self-Determination Theory [39], the flexibility of remote learning may have allowed some adolescents to exercise greater autonomy. Some may also have experienced a greater sense of competence while working at home. Additionally, some adolescents may have experienced closer relationships with family members, enhancing their sense of relatedness. A greater satisfaction of the needs for autonomy, competence, and relatedness is linked to better well-being [39] and may be reflected in a decrease in conduct problems and hyperactivity-inattention problems. Moreover, reduced peer interactions may have prevented some adolescents from engaging in problem behaviors, such as fighting, and the home environment may have afforded fewer distractions than the classroom, leading to improved concentration.” (p. 24-25)

7. Discussion: Further exploration of why girls were overrepresented across multiple trajectories (e.g., increasing, decreasing, and high-risk) could add nuance. Exploring how gender roles or coping mechanisms may have influenced these results would provide valuable insight.

Response: This comment suggests that our original text aimed to explain the gender differences was not clear enough. Therefore, we rephrased the relevant section in the Discussion to underline that gender itself is not the driver of increases or decreases in mental health problems during the pandemic.

“Girls were overrepresented in the increasing-risk, decreasing-risk, and high-risk groups of emotional symptoms, while boys were overrepresented in the decreasing-risk and high-risk groups of conduct problems. Previous research has shown that girls reported greater increases in internalizing problems than boys during the pandemic [18]. However, our findings suggest that gender did not necessarily drive vulnerability to emotional symptoms during the pandemic. This is evident from the overrepresentation of girls not only in the increasing-risk group but also in the group showing a decrease in emotional symptoms. Similarly, boys were overrepresented in the trajectories with the highest pre-pandemic levels of conduct problems, whether these problems remained stable or decreased during the pandemic. In line with existing research on gender differences in internalizing and externalizing mental health problems [17], these results suggest that gender is more strongly associated with pre-pandemic levels of emotional and conduct problems than with the trajectories of these problems during the pandemic.” (p. 28)

8. Discussion: The inconsistent associations between demographic characteristics (e.g., migration background, SES) and trajectories warrant further exploration. While the findings that these variables do not systematically predict mental health outcomes are noted, a more nuanced discussion could elucidate why this might be the case, considering differences in school closures, family dynamics, or cultural factors.

Response: We added a paragraph to the discussion in which we explain why we may not have found systematic differences for migration background and family SES.

“Thus, neither having a migration background nor coming from a lower SES family should be considered direct risk factors for developing or experiencing higher levels of mental health problems during the COVID-19 pandemic. Adolescents with a migration background or from lower SES families are possibly too heterogeneous, with diverse experiences and circumstances that may more directly influence their mental health. Factors such as parental employment in essential sectors (e.g., healthcare) or living in crowded homes with limited opportunities for remote work or effective study can exacerbate stress within families, potentially impacting adolescent mental health. Moreover, adolescent subjective SES is more strongly associated with adolescent mental health problems than family SES and can even buffer the association between family SES and mental health problems [41]. Thus, factors such as parental employment type, living arrangements, and subjective SES may be better able to predict which adolescents experienced changes in mental health problems during the pandemic than migration background or family SES.” (p. 27)

9. Discussion: While the discussion highlights some non-significant findings (e.g., no systematic link between demographic factors and mental health trajectories), it could further explore why expected patterns, such as migration background or SES predicting vulnerability, did not emerge. Considering whether the pandemic created conditions that levelled typical social or demographic disparities would add depth to the interpretation.

Response: We thank Reviewer 1 for this interesting suggestion. In response, we have added a paragraph in the Discussion section where we considered the possibility that the pandemic may have created conditions that leveled typical demographic or social disparities in mental health outcomes.

“Furthermore, our results suggest that the COVID-19 pandemic has created unique conditions that temporarily leveled typical demographic or social disparities in mental health outcomes. For instance, widespread disruptions and shared experiences of uncertainty, social isolation, and restrictions could have overridden individual differences such as having a migration background or growing up in lower SES families, which are associated with a greater vulnerability to mental health problems. This aligns with the idea that strong situations constrain behavioral options and limit variability across individuals [42]. In this sense, the COVID-19 pandemic could be considered a strong situation in which personal characteristics may have been less influential on mental health outcomes than in a context outside the pandemic.” (p. 27-28)

**Reviewer 2**

SUMMARY

The article examines the trajectories of mental health problems in adolescents before and during the COVID-19 pandemic. Using data from 1,522 adolescents collected at four different points between 2019 and 2021, the study identifies various trajectories of emotional symptoms, conduct problems, hyperactivity, and peer relationship issues. Four main trajectories were identified for emotional symptoms, conduct problems, and hyperactivity: stable low risk, stable high risk, increasing low risk, and decreasing moderate risk. For ADHD, 5th trajectory was found: increasing moderate risk. For peer relationship problems, two trajectories were found: stable low and stable high.

The findings show that the pandemic did not affect all adolescents in the same way. Some experienced increasing symptoms (especially those with low starting values), others saw decreases, while the majority remained stable (at both a high and low level). Adolescents with high levels of mental health problems before the pandemic also reported relatively low levels of family and peer support. Demographic characteristics such as gender, migration background and socioeconomic status were not consistently linked to specific trajectories. Overall, these results emphasize the need for tailored intervention strategies to meet the specific needs of different groups of adolescents during stressful times.

GENERAL COMMENT

The subject is very interesting, and the authors have partly dealt with it in the appropriate way. Of note is also the high quality of the data. This article is therefore of interest to the psychopathology research community as well as to the public health research community that might compose the broad readership of Plos Mental Health. The introduction is clear and well-documented, explaining both the challenges and the strengths of the study. On the latter point, the authors have a larger sample size than other previous studies, measures of mental health that have been widely validated internationally (internalizing/externalizing problems) and collected at different points in time (before, during and after the pandemic), and socio-demographic data that are relevant for studying inter-individual variability within classes. The objectives and hypotheses are also set out very clearly. I found the discussion objective, avoiding over-interpretation. Finally, the statistical framework used by the authors is appropriate for meeting their first objective (Identifying different trajectories of adolescent mental health problems before and during the COVID-19 pandemic, using a sizeable, prospective, longitudinal study). However, I have a major concern with the way the authors described their analytic choices (which often lack justification), and also with the method they chose to achieve their second objective (exploring whether migration background, family SES, gender, family support, and friend support were associated with the identified trajectories). I think the authors have every opportunity to address these concerns, and I hope I can help them in this endeavor. Overall, I think that the methodological choices made by the authors to study the time series must be better justified. I also think that the comprehension of the latent class model’s results on the light of auxiliary predictors could greatly benefit from an alternative approach. To summarize, this article has the potential to be published in Plos Mental Health. I hope that the authors will find in my few comments an opportunity to improve the quality of their manuscript.

Response: We would like to thank Reviewer 2 for the positive comments on the introduction, discussion, and the valuable, concrete, and helpful suggestions, especially on the analytical part. Below we have addressed the comments, and we are confident that the revisions have improved the quality of the manuscript.

MAJOR

1. I would recommend the authors to follow the guidelines for reporting on latent trajectories study by van de Schoot et al (van de Schoot R, Sijbrandij M, Winter SD, Depaoli S, Vermunt JK. 2016 The GRoLTS-checklist: guidelines for reporting on latent trajectory studies. Struct Equat Model 24, 451-467, doi:10.1080/10705511.2016.1247646). The purpose of this paper is to present criteria that should be included when reporting the results of latent trajectory analysis across research fields. It summarizes in a very comprehensive way several important decisions that researchers must make while writing the results of their study (for example, why choosing a LGCA model instead of a LGMM model?).

I would ask authors to check carefully whether they complete all the points that might concern them, and to include in the revised version of the manuscript any changes (analytic, semantic, graphical, etc) that might result from this examination.

Response: We thank Reviewer 2 for bringing these guidelines to our attention. We checked the items of the guidelines and processed most of them into the manuscript. We added the following information to the manuscript:

- “We selected LCGA over latent growth mixture modeling because our primary interest was to identify subgroups with similar patterns, rather than to model individual differences within those subgroups.” (p. 10)
- “The intervals between time points were adjusted to correspond with the time period between the measurements (i.e., the time points for the linear slope factor were fixed at 0, 0.5, 1, and 2). We used starting values 100 for the single-class growth models.” (…) “We used starting values 100 10 for these models. If we encountered computation problems, we increased the LRTSTARTS to 500 100 250 50.” (p. 11)
- “To handle missing data without deleting cases and to limit the bias associated with missing data and non-normality, we used maximum likelihood with robust standard errors (MLR) [29].” (p. 12)
- “The dataset and syntax are available on the Open Science Framework (OSF, https://osf.io/j2t6w/). Additionally, plots displaying the estimated mean trajectories for each model, as well as plots showing the estimated means of the final model and the observed individual trajectories for each latent class, are available on OSF.” (p. 11)

Additionally, we added information about the standard deviations and sample size to Table 3.

Item 8, regarding how covariates were added to the model, is described in more detail under Comment 2.

2. Related to this, I think that the comprehension of the latent class model’s results on the light of auxiliary predictors could greatly benefit from a more elaborated methodology. The authors propose to study whether gender, migration background, family support, friend support, and family socio-economic status, have an impact on the distribution of subjects between the estimated classes. To do this, they look at each class separately (eg, increasing low-risk) to see if subjects differ in each of the factors listed above.

I see some limitations to this approach. First, there is the use of multiple chi-square tests to understand the structure of each class whose significance threshold, as far as I understand, is not adjusted for this multiple testing. Second, beyond the fact that this approach hampers the overall understanding of the results, its main problem is that it treats classes as (real) observed objects, whereas they are merely estimated objects involving subjects classified with more or less statistical precision. Clark & Muthen explain this problem very clearly in their methodological article « Relating Latent Class Analysis Results to Variables not Included in the Analysis » (<http://www.statmodel.com/download/relatinglca.pdf>):

« Suppose a 2-class model and take two individuals, one with a probability of 1.0 for belonging to Class 1 and 0.0 for Class 2 and the other with a probability of 0.51 for belonging to Class 1 and 0.49 for Class 2. Both individuals would be assigned and treated as members of Class 1 in the subsequent analyses. But the analyses does not take into account that the two individuals have different probabilities of being in the same class and instead are treated as if they both have a probability of 1.0 of being in Class 1. This will distort estimates because individuals are forced into their most likely latent classes. The standard errors will also be incorrect because the analysis does not take into account the uncertainty of the classification but treats it as an observed variable. This poses a problem because incorrect standard errors can lead to erroneous conclusions about the significance of an effect. »

These difficulties can be mitigated by using a three-step approach, again well described in the van de Schoot paper. I would like to encourage the authors to apply such an approach. The 1st step determines the number of latent classes without the predictors on class membership.

The 2nd step simply consists in saving the most likely class membership and related probability (classification precision), and then merged it with the original data. The 3rd step consists in analysing class membership separately from the latent trajectory model using a (univariate and then backward stepwise) multinomial regression analysis weighted for the posterior probability of class membership (hence accounting for classification imprecision). The authors may eventually dig even further by investigating more precisely how the probability of being assigned into a given class of symptoms trajectory varies for each level of a predictor of interest (eg, gender, family SES).

A practical application of this approach can be found in:

Edjolo A, Dartigues JF, Pérès K, Proust-Lima C. (2020). Heterogeneous long-term trajectories of dependency in older adults: the PAQUID cohort, a population-based study over 22 years. J. Gerontol Med Sci 75, 2396-2403. (doi:10.1093/gerona/glaa057)

or in:

Safra, L., Lettinga, N., Jacquet, P. O., & Chevallier, C. (2022). Variability in repeated economic games: comparing trust game decisions to other social trust measures. R Soc Open Sci, 9(9), 210213 (<https://doi.org/10.1098/rsos.210213>)

Response: We would like to thank Reviewer 2 for raising this point and for the detailed explanation, including references and concrete suggestions. We fully agree with the reviewer that it is essential to account for the uncertainty of classification, and we have already applied this approach in our analyses. Specifically, we used the three-step approach as described in: Asparouhov T, Muthén B. Auxiliary variables in mixture modeling: Three-step approaches using Mplus. Structural Equation Modeling. 2014;21:329–341. doi:10.1080/10705511.2014.915181.

We realized that the article did not adequately describe the method we used to include the predictors. Therefore, we now provide a more detailed explanation of this in the Method section:

“After estimating the best fitting model and establishing the number of classes, we used a three-step approach to examine the extent to which the identified classes differed from each other on the demographic and social support variables [27]. First, the latent class model was estimated. Second, the most likely class variable was created using the latent class posterior distribution. Third, we added the demographic and social support variables one by one and tested the equality of means or percentages across classes using chi-square tests. In this third step, misclassification from the second step was taken into account.” (p. 11-12)

MINOR

3. The authors report that a significant proportion of teens saw their problems increase or decrease during the pandemic. It would be interesting to put the scores observed for the “increasing“ and the “decreasing“ classes of subjects at the end of the pandemic into perspective with the severity thresholds identified by epidemiological studies on SDQ. For example, if we refer to the reference studies used by SDQ designers to establish severity cut-offs (<https://www.sdqinfo.org/a0.html>), it appears that the scores of teens belonging to the “increasing” groups remain within non-problematic limits. The only “increasing“ score that appears to be “on the edge“ is the Emotional symptoms score. Considering this, a question can be addressed: To what extent can the “changes” identified by the latent class model be considered as genuine changes rather than random fluctuations around the mean? This possibility is already raised by the authors in the discussion, but it might be interesting to elaborate more on it.

Response: Thank you for this suggestion. We used the cut off scores of Vugteveen et al. (2022) for Dutch self-reported SDQ for age 17, and have incorporated this into the Method, Results, and Discussion.

Method: “To interpret the scores obtained from the SDQ-R, we applied cutoff values established using normative data for Dutch 17-year-old adolescents [24]. These cutoffs categorize scores into three groups: ‘normal’, ‘borderline’, and ‘abnormal’. Adolescents with scores at or above the 90th percentile were classified as ‘abnormal’, representing those with the most extreme scores. Adolescents scoring in the next 10%, corresponding to the 80th percentile, were categorized as ‘borderline’. For emotional symptoms, scores of six or higher were considered ‘abnormal’, and five ‘borderline’. For conduct problems, scores of three or higher were ‘abnormal’, and two ‘borderline’. For hyperactivity-inattention problems, scores of seven or higher were ‘abnormal’, and six ‘borderline’. For peer relationship problems, scores of four or higher were ‘abnormal’, and three ‘borderline’.” (p. 9)

In the Result section, we added interpretation of the scores for the trajectories of the different mental health problems, including sentences such as: “Emotional symptoms in both the increasing-risk and decreasing-risk groups remained within the ‘normal’ range.” and “While the low-risk and increasing low-risk groups remained in the ‘normal’ range, the increasing moderate-risk group shifted from ‘normal’ to ‘abnormal’.”

Discussion: “Using the SDQ cutoff values based on normative data for Dutch 17-year-old adolescents [24], emotional symptoms in both the increasing-risk and decreasing-risk groups remained within the ‘normal’ range. This suggests that the COVID-19 pandemic did not have a substantial impact on adolescent emotional symptoms. Conduct problems may have been affected more by the pandemic, as the increasing-risk group shifted from the ‘normal’ to ‘borderline’ range, while the decreasing-risk group moved from ‘borderline’ to ‘normal’. The pandemic seems to have had the largest impact on hyperactivity-inattention problems, as the most pronounced changes were observed for this subscale. The moderate increasing-risk group shifted from ‘normal’ to ‘abnormal’, while the decreasing-risk group moved from ‘abnormal’ to ‘normal’.” (p. 22-23)

4. The SDQ also has a functional impact supplement. Was this supplement administered, and if yes, why was it not the object of temporal analyses?

Response: Unfortunately, the functional impact supplement of the SDQ was not administered in this study, so we could not include it in the analyses.
